# Supplementary material for: High-molecular-weight polymers from dietary fiber drive aggregation of particulates in the murine small intestine
Source: eLife. 2019 Jan 22;8:e40387. doi: 10.7554/eLife.40387 (PMC6342521; doi:10.7554/eLife.40387)
Supplement: Transparent reporting form [file elife-40387-transrepform.pdf]

eLIFE
1st Floor
24 Hills Road
Cambridge CB2 1JP, UK
P 01223 855340
W elifesciences.org
T @elife

eLife's transparent reporting form

We encourage authors to provide detailed information *within their submission* to facilitate the interpretation and replication of experiments. Authors can upload supporting documentation to indicate the use of appropriate reporting guidelines for health-related research (see EQUATOR Network), life science research (see the BioSharing Information Resource), or the ARRIVE guidelines for reporting work involving animal research. Where applicable, authors should refer to any relevant reporting standards documents in this form.

If you have any questions, please consult our Journal Policies and/or contact us: editorial@elifesciences.org.

Sample-size estimation

- You should state whether an appropriate sample size was computed when the study was being designed
- You should state the statistical method of sample size computation and any required assumptions
- If no explicit power analysis was used, you should describe how you decided what sample (replicate) size (number) to use

Please outline where this information can be found within the submission (e.g., sections or figure legends), or explain why this information doesn't apply to your submission:

No explicit power analysis was used because these experiments rely on analyzing the sizes of aggregates of particles where the total number of aggregates in solution varies among samples. All experiments had at least 300 aggregates in solution, in many cases containing ~1000 or more aggregates. To compute 95% empirical bootstrap confidence intervals of the average aggregate size, which were used to draw comparisons between samples, we generated 10,000 bootstrap replicates from the original empirical cumulative distribution functions of each sample. While there is not a standard method to determine the appropriate number of bootstrap replicates for these types of samples, we found that there were only small variations between confidence intervals computed with 100 and 1,000 replicates. Because we had sufficient computational power, we increased the number of bootstrap replicates to 10,000.

Replicates

- You should report how often each experiment was performed
- You should include a definition of biological versus technical replication
- The data obtained should be provided and sufficient information should be provided to indicate the number of independent biological and/or technical replicates
- If you encountered any outliers, you should describe how these were handled
- Criteria for exclusion/inclusion of data should be clearly stated
- High-throughput sequence data should be uploaded before submission, with a private link for reviewers provided (these are available from both GEO and ArrayExpress)

Please outline where this information can be found within the submission (e.g., sections or figure legends), or explain why this information doesn't apply to your submission:

eLife Sciences Publications, Ltd is a limited liability non-profit non-stock corporation incorporated in the State of Delaware, USA, with company number 5030732, and is registered in the UK with company number FC030576 and branch number BR015634 at the address 1st Floor, 24 Hills Road, Cambridge CB2 1JP | August 2014

All experiments with small intestinal luminal fluid (and colonic luminal fluid for Fig 5E) were performed with samples that were pooled from groups of three mice of the same age that were co-housed, as explained in detail in Materials and Methods ("Collection of intestinal luminal fluid"). We used pooled samples from groups of three mice because the luminal content from an individual mouse was insufficient in volume to perform all the required analyses (i.e. *ex vivo* aggregation, GPC, and sometimes Western blot). As described in Fig 2, we found that there was little variability among pooled samples from groups of mice that were treated under the same conditions (i.e. same strain, same diet, same housing conditions, same gender, similar age). When 95% bootstrap confidence intervals were generated, we created 10,000 bootstrap replicates, as described in Materials and Methods ("Quantification of aggregate sizes").

Statistical reporting

- Statistical analysis methods should be described and justified
- Raw data should be presented in figures whenever informative to do so (typically when N per group is less than 10)
- For each experiment, you should identify the statistical tests used, exact values of N, definitions of center, methods of multiple test correction, and dispersion and precision measures (e.g., mean, median, SD, SEM, confidence intervals; and, for the major substantive results, a measure of effect size (e.g., Pearson's r, Cohen's d)
- Report exact p-values wherever possible alongside the summary statistics and 95% confidence intervals. These should be reported for all key questions and not only when the p-value is less than 0.05.

Please outline where this information can be found within the submission (e.g., sections or figure legends), or explain why this information doesn't apply to your submission:

Statistical analysis is described in detail in Materials and Methods, specifically "Quantification of aggregate sizes". As described in Materials and Methods, the advantage of bootstrapping to generate 95% empirical bootstrap confidence intervals is that we do not need to assume anything about the underlying probability distribution that governs aggregate formation; the approach is non-parametric.

(For large datasets, or papers with a very large number of statistical tests, you may upload a single table file with tests, Ns, etc., with reference to sections in the manuscript.)

Group allocation

- Indicate how samples were allocated into experimental groups (in the case of clinical studies, please specify allocation to treatment method); if randomization was used, please also state if restricted randomization was applied
- Indicate if masking was used during group allocation, data collection and/or data analysis

Please outline where this information can be found within the submission (e.g., sections or figure legends), or explain why this information doesn't apply to your submission:

For mouse groups, each group of pooled mice always consisted of three, co-housed mice of the same strain, gender, and age. Mice of the same genetic background were randomly allocated into groups. No masking was used.

Additional data files ("source data")

- We encourage you to upload relevant additional data files, such as numerical data that are represented as a graph in a figure, or as a summary table
- Where provided, these should be in the most useful format, and they can be uploaded as “Source data” files linked to a main figure or table
- Include model definition files including the full list of parameters used
- Include code used for data analysis (e.g., R, MatLab)
- Avoid stating that data files are “available upon request”

Please indicate the figures or tables for which source data files have been provided:

Source data, Python code for statistical analyses, and ImageJ macros used for image analysis have been deposited in Dryad’s database.
